# Sex difference in outcomes after coronary artery bypass grafting: follow-up data of the Netherlands Heart Registration

**DOI:** 10.1007/s12471-024-01920-5

**Published:** 2024-12-16

**Authors:** Mara-Louise Wester, Jules R. Olsthoorn, Mohamed A. Soliman-Hamad, Saskia Houterman, Maaike M. Roefs, Joost F. J. ter Woorst, Cardiothoracic Surgery Registration Committee of the Netherlands Heart Registration, S. Bramer, W. J. P. van Boven, A. B. A. Vonk, B. M. J. A. Koene, J. A. Bekkers, G. J. F. Hoohenkerk, A. L. P. Markou, A. de Weger, P Segers, F. Porta, R. G. H. Speekenbrink, W. Stooker, W. W. L. Li, E. J. Daeter, N. P. van der Kaaij, G. Vigano

**Affiliations:** 1 Department of Cardiothoracic Surgery, Catharina Hospital, Eindhoven, The Netherlands; 2 Department of Education and Research, Catharina Hospital, Eindhoven, The Netherlands; 3 Netherlands Heart Registration, Utrecht, The Netherlands

**Keywords:** Coronary artery bypass grafting, Male–female differences, Nationwide registry

## Abstract

**Objectives:**

Controversies exist regarding sex differences in outcomes after coronary artery bypass grafting (CABG). This study assessed sex differences in early and mid-term outcomes after CABG and factors associated with these differences. Outcomes were based on data from the Netherlands Heart Registration (NHR).

**Methods:**

Data of patients undergoing CABG in the Netherlands between 2013 and 2019 were retrieved from the NHR database. Primary outcomes were early mortality, morbidity and mid-term survival. The population was divided into subgroups based on age (≥ 70 years and < 70 years). Regression analyses investigated the correlation between sex and both early and mid-term mortality.

**Results:**

This study included 41,705 male and 10,048 female patients. Median follow-up was 3.6 (1.8–4.8) years. Female patients were less likely to receive ≥ 2 arterial grafts (15.9% vs 23.2%, *p* < 0.001), had fewer anastomoses (3.2 ± 1.1 vs 3.5 ± 1.1, *p* < 0.001), higher 30-day mortality (1.9% vs 1.0%; *p* < 0.001) and a lower mid-term survival rate (91.3% vs 93.1%, *p* < 0.001). Perioperative complications, including myocardial infarction and stroke, were more common in female patients (all *p* < 0.001). Women aged < 70 years had a lower mid-term survival rate than men < 70 years (94.5% vs 96.0%, *p* < 0.001). Cox regression analysis showed that female sex was not significantly associated with mid-term mortality in the total cohort [hazard ratio (HR) 1.03; *p* = 0.45] but was associated with mid-term mortality in patients aged < 70 years (HR 1.19; *p* < 0.001).

**Conclusions:**

Women undergoing CABG in our cohort presented with more complex risk profiles, received different surgical strategies and had worse early and mid-term outcomes compared to men. Female sex was associated with mid-term mortality only in patients < 70 years of age.

**Supplementary Information:**

The online version of this article (10.1007/s12471-024-01920-5) contains supplementary material, which is available to authorized users.

## What’s new?

- Female patients undergoing coronary artery bypass grafting (CABG) continue to have worse 30-day and mid-term mortality.
- This large contemporary study in the Netherlands confirms that women undergoing CABG have a more complex preoperative risk profile than men.
- The surgical technique for female patients is different to that for male patients.
- Women receive fewer distal anastomoses and less frequently receive ≥ 2 arterial grafts.
- Female sex was significantly associated with mid-term mortality in patients < 70 years of age.

## Introduction

Coronary artery bypass grafting (CABG) remains the cornerstone in the treatment of complex coronary artery disease (CAD) [1]. Both early and late mortality after CABG have decreased over time [2, 3]. However, the outcomes after CABG in women differ from those in men. Early mortality (30-day, in-hospital mortality) after CABG has even been reported to be twice as high in women compared to men [1–3].

Female sex is included as a risk factor in both the European System for Cardiac Operative Risk Evaluation (EuroSCORE) algorithm and the Society for Thoracic Surgeons (STS) risk model [4, 5]. However, it is still a matter of debate in the literature with arguments stating that differences in outcomes can be explained by clustering of cardiovascular risk factors and more complex clinical presentation in women, and counterarguments stating that female sex itself was associated with increased mortality and major adverse cardiovascular events after adjustment for these risk factors [6, 7]. If the former is the case, and it is not female sex itself that leads to a different outcome, sex could be reconsidered as a stand-alone factor in the current risk scores concerning outcomes of cardiac surgery (EuroSCORE and STS score) [4].

**Fig. 1:**
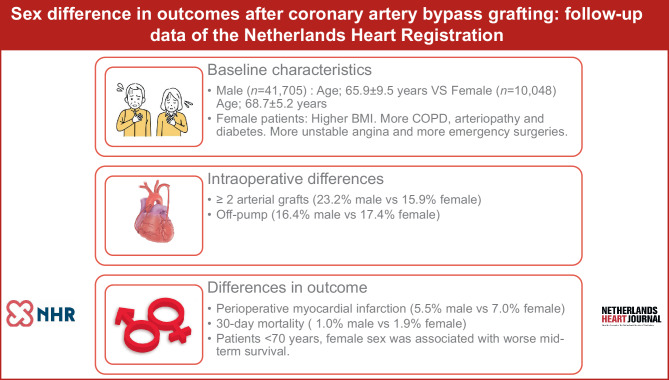
Infographic: Coronary artery bypass surgery in the Netherlands. *BMI* body mass index, *COPD* chronic obstructive pulmonary disease

Recent large studies have demonstrated that there is a higher incidence of major adverse cardiac and cerebrovascular events after coronary artery surgery among female patients compared to male patients [6]. Furthermore, perioperative complications weigh differently between sexes, indicating that if men and women suffer from the same complication, women appear to have worse outcomes [2]. The aim of this study was to assess differences in early and mid-term outcomes between men and women undergoing CABG in the Netherlands, thereby determining the factors which contribute to these differences in outcomes (Infographic: Fig. 1).

## Methods

### Study population

This study included data from the national database of the Netherlands Heart Registration (*Nederlandse Hart Registratie*, NHR). This prospective database comprises data of all patients undergoing cardiac surgery in the Netherlands. The process and rationale behind data collection was reported elsewhere [8]. In addition to demographic characteristics and type of intervention, the database also includes parameters concerning postoperative morbidity and mortality and all risk factors that are required for calculation of the most frequently used risk scores. Although this database was initiated for quality evaluation of cardiovascular interventions, its completeness in registering pre- and postoperative variables enables anonymised long-term follow-up of patients undergoing cardiac surgical interventions. For this study, isolated CABG or off-pump coronary artery bypass grafting (OPCAB) cases were included for analysis. The Appendix lists all hospitals that participate in the registry.

### Design and ethical statement

The study was a retrospective multi-centre cohort study of prospectively collected data from 16 cardiothoracic centres in the Netherlands. Data were collected for patients undergoing CABG between 2013 and 2019. The Medical Research Ethics Committee (MEC-U) approved the study and waived the need for informed consent (W19.269).

### Outcomes

The NHR database was searched for all preoperative data and baseline characteristics required for calculation of the EuroSCORE I [4]. EuroSCORE II was not calculated, as some values required to calculate this version of the EuroSCORE were not available [4]. Short-term outcomes included early mortality (30-day mortality), postoperative complications [stroke, myocardial infarction (MI), pneumonia, reintubation, prolonged ventilation, readmission to the intensive care unit, renal failure, gastrointestinal complications, re-exploration for bleeding and arrhythmia] and length of hospital stay. Definitions of postoperative complications are provided in Tab. S1 (Electronic Supplementary Material).

During mid-term follow-up, all-cause mortality was derived from the municipal administration records and was completed for all patients. We defined reintervention as unplanned coronary intervention of the target coronary vessel, including percutaneous coronary intervention (PCI) or CABG, after initial treatment. If patients underwent both PCI and CABG postoperatively, the first event that occurred counted. Reinterventions did not include planned PCI as a part of hybrid coronary revascularisation following primary CABG.

### Missing values

The NHR database has an exceptionally high data completeness for baseline characteristics. Two non-mandatory variables had a relatively high proportion of missing data: extracorporeal circulation time (ECC time) (18.2%) and cross-clamp time (18.1%). Given the higher levels (> 10%) of missing data for these variables, it was inappropriate to apply a multiple imputation method. Furthermore, 118 patients (0.2%) were lost to follow-up and therefore were not included in survival analysis.

### Statistical analysis

Continuous variables are presented as mean ± SD, in case of skewedness as median with interquartile range. Categorical data are expressed as frequencies and percentages and were compared using the χ^2^ test. If the minimum expected cell-size assumption did not apply, data were analysed with Fisher’s exact test. Continuous variables were compared using a *t*-test or, in the case of non-normal distribution, with the Mann-Whitney U test. Mid-term survival was demonstrated with Kaplan-Meier survival curves. Subgroup analysis was performed for patients aged < 70 years and ≥ 70 years, based on the recommendation of the current guidelines for conduit selection [8]. Differences between survival curves were assessed using the log-rank test. Variables that were associated with mid-term mortality were identified using Cox regression. Independent variables with a significance level *p* < 0.05 in the univariate model were entered in the multivariate model. Proportional hazard assumption was checked graphically using log minus log survival plots. Hazard ratios (HR) are reported with associated 95% confidence intervals (CI). All reported *p-*values were two-sided and were considered statistically significant if *p* < 0.05. Statistical analyses were performed using SPSS software (V26, IBM, Armonk, NY, USA) and R statistics (R Foundation, Vienna, Austria).

## Results

### Baseline characteristics

In total, 51,753 patients underwent CABG in the Netherlands between 2013 and 2019. The majority of patients were male (80.6%); the mean age of the study population was 66.4 ± 9.5 years. Comorbidities, including diabetes mellitus (DM), chronic lung disease and peripheral vascular disease (PVD), were more common in women than in men. Furthermore, unstable angina pectoris was more frequently observed in women, and consequently, emergency surgery was performed more often in women than in men. These differences resulted in a higher median logistic EuroSCORE for women [4.30 (2.47–7.58)] compared to men [2.53 (1.51–4.66)] (*p* < 0.001). Further demographic and baseline characteristics are shown in Table 1, and Table S2 (Electronic Supplementary Material) shows the baseline characteristics of the age subgroups (< 70 and ≥ 70 years).

**Table 1:** Baseline characteristics

	Total cohort	Male	Female	*p*-value
	*N* = 51,753	*n* = 41,705 (80.6%)	*n* = 10,048 (19.4%)	
Age, years	66.4 ± 9.5	65.9 ± 9.5	68.7 ± 5.2	*<* *0.001*
BMI > 25 kg/m^2^	37,973 (37.4)	27,925 (67.0)	10,048 (100.0)	*<* *0.001*
BSA, m^2^	1.99 (1.86–2.10)	2.01 (1.92–2.14)	1.79 (1.69–1.92)	*<* *0.001*
Chronic lung disease	4,996 (9.7)	3,880 (9.3)	1,116 (11.1)	*<* *0.001*
Extracardiac arteriopathy	6,111 (11.8)	4,825 (11.6)	1,286 (12.8)	* 0.001*
Diabetes	13,287 (25.7)	10,119 (24.3)	3,168 (31.5)	*<* *0.001*
Serum creatinine, μm/l	86 (74–99)	88 (78–101)	73 (63–86)	*<* *0.001*
Unstable angina	4,726 (9.1)	3,582 (8.6)	1,144 (11.4)	*<* *0.001*
Recent myocardial infarction	16,826 (32.5)	13,496 (32.4)	3,330 (33.1)	0.13
Emergency	3,125 (6.0)	2,355 (5.6)	770 (7.7)	*<* *0.001*
LV function				
– Good	35,045 (67.7)	27,851 (66.8)	7,194 (71.6)	
– Moderate	13,305 (25.7)	11,039 (26.5)	2,266 (22.6)	
– Poor	1,748 (3.4)	1,467 (3.5)	281 (2.8)	
– Very poor	369 (0.7)	304 (0.7)	65 (0.6)	
Prior cardiac surgery, *n* (%)	1,093 (2.1)	879 (2.1)	214 (2.1)	0.89
EuroSCORE I, median (IQR)	2.79 (1.57–5.13)	2.53 (1.51–4.66)	4.30 (2.47–7.58)	*<* *0.001*

Data are presented as mean ± SD, median (IQR) or *n* (%). The *p*-value stands for differences in characteristics

*BMI* body mass index, *BSA* body surface area, *LV* left ventricular, *IQR* interquartile range

### Operative characteristics

OPCAB was performed more frequently in women than in men (17.4% vs 16.4%, *p* = 0.02). The number of distal anastomoses was higher among men than among women [median 3.0 (3.0–4.0) vs 3.0 (2.0–4.0), *p* < 0.001]. More men than women received ≥ 2 arterial grafts (23.2% vs 15.9%, *p* < 0.001). Additional operative details are shown in Tab. 2.

**Table 2:** Operative characteristics and postoperative complications

*Operative characteristics*	Total cohort	Male	Female	*p-*value
	*N* = 51,753	*n* = 41,705	*n* = 10,048	
Off-pump CABG	8,605 (16.6)	6,853 (16.4)	1,752 (17.4)	* 0.02*
Number of anastomoses	3.0 (3.0–4.0)	3.0 (3.0–4.0)	3.0 (2.0–4.0)	*<* *0.001*
Type of conduit				*<* *0.001*
Single-artery graft	3,738 (7.2)	2,834 (6.8)	904 (9.0)	
Left internal mammary artery and vein graft	35,108 (67.8)	28,011 (67.2)	7,097 (70.6)	
Multiple arterial	2,218 (4.3)	1,959 (4.7)	259 (2.6)	
Total arterial	9,040 (17.5)	7,703 (18.5)	1,337 (13.3)	
Vein graft only	1,649 (3.2)	1,198 (2.9)	451 (4.5)	
≥ 2 arterial grafts	11,258 (21.8)	9,662 (23.2)	1,596 (15.9)	*<* *0.001*
*Postoperative complications*				
Hospital stay, days	5.0 (4.0–6.1)	5.0 (4.0–6.1)	5.0 (4.0–7.0)	*<* *0.001*
30-day mortality	611 (1.2)	420 (1.0)	191 (1.9)	*<* *0.001*
Myocardial infarction	3,008 (5.8)	2,309 (5.5)	699 (7.0)	*<* *0.001*
Arm/leg wound infection	304 (0.6)	227 (0.5)	77 (0.8)	* 0.01*
Deep sternal wound infection	451 (0.9)	332 (0.8)	119 (1.2)	*<* *0.001*
Pneumonia	1,453 (2.8)	1,242 (3.0)	211 (2.1)	*<* *0.001*
Respiratory insufficiency	755 (1.5)	613 (1.5)	142 (1.4)	0.63
Prolonged intubation (> 24 h)	1,150 (2.2)	875 (2.1)	275 (2.7)	*<* *0.001*
Readmission to ICU	1,229 (2.4)	980 (2.3)	249 (2.5)	0.48
Stroke	481 (0.9)	356 (0.9)	125 (1.2)	*<* *0.001*
Arrhythmia	12,470 (24.1)	10,070 (24.1)	2,400 (23.9)	0.51
Re-exploration	1,893 (3.6)	1,602 (3.8)	291 (2.9)	*<* *0.001*

Data are presented as median (IQR) or *n* (%)

The *p*-value stands for differences in characteristics

*CABG* coronary artery bypass grafting, *ICU* intensive care unit, *IQR* interquartile range

### Outcomes

In the overall cohort (*n* = 51,753), 30-day mortality was 1.2% (*n* = 611), and perioperative MI occurred in 5.8% of patients. A higher 30-day mortality rate was observed in women than in men (1.9% vs 1.0%, *p* < 0.001). The incidence of perioperative MI, stroke, prolonged intubation, urinary tract infection (UWI) and deep sternal wound infection was higher in women than in men (all *p* < 0.001) (Tab. 2).

**Fig. 2:**
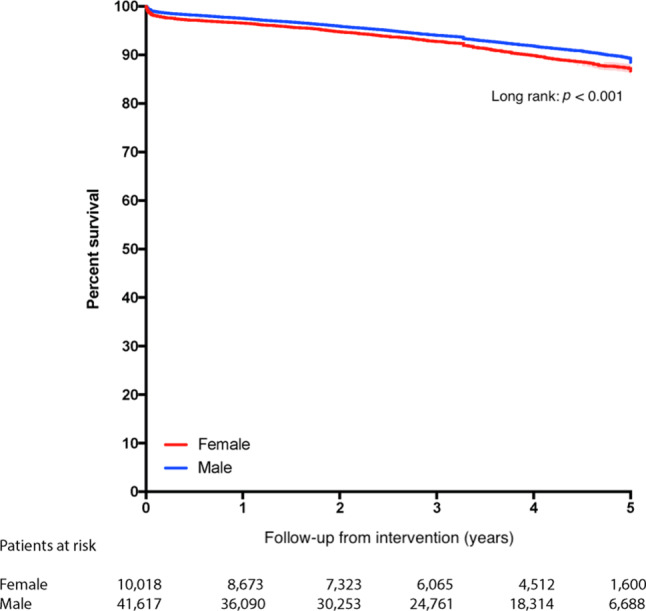
Kaplan-Meier survival curve: total population

**Fig. 3:**
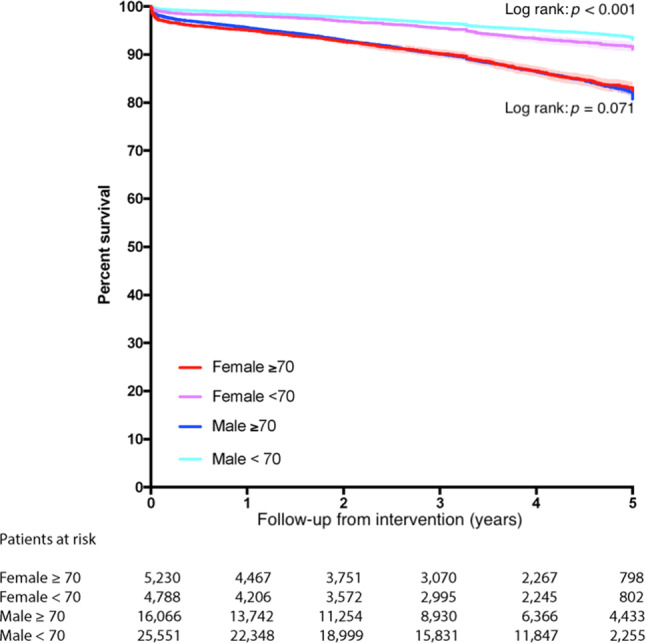
Kaplan-Meier survival curve: age groups

Median follow-up for the total cohort was 3.6 (1.8–4.8) years [men 3.5 (1.8–4.8) years, women 3.6 (1.7–4.8)]. In the total cohort, mid-term survival rate was 92.7% (93.1% in male patients, 91.3% in female patients (*p* < 0.001)) (Fig. 2). For patients aged ≥ 70 years, no significant difference in mid-term survival was observed between men and women (*p* = 0.71) (Fig. 3). In the subgroup analysis for patients aged < 70 years, a statistically significant worse mid-term survival was found in women compared to men (*p* < 0.001) (Fig. 3).

Multivariate Cox regression analysis (Table S3, Electronic Supplementary Material; Tab. 3) revealed that female sex itself was not significantly associated with mid-term mortality (Table S3, Electronic Supplementary Material). In patients aged < 70 years (Tab. 3), worse mid-term survival was associated with female sex, age, chronic lung disease, PVD, DM, serum creatinine > 200 µm/l, recent MI, decreased left ventricular ejection fraction (LVEF) and emergency surgery. Both OPCAB and the use of ≥ 2 arterial grafts were associated with lower mid-term mortality. In the subgroup ≥ 70 years (Tab. 3), no statistically significant association was found between mid-term mortality and female sex (Tab. 3).

**Table 3:** Cox regression analysis for mid-term mortality in the subgroups < 70 years and ≥ 70 years

	Univariate < 70		Multivariate < 70		Univariate ≥ 70		Multivariate ≥ 70	
	HR (95% CI)	*p*-value	HR (95% CI)	*p*-value	HR (95% CI)	*p*-value	HR (95% CI)	*p*-value
Age	1.07 (1.06–1.08)	*<* *0.001*	1.06 (1.05–1.07)	*<* *0.001*	1.09 (1.08–1.10)	*<* *0.001*	1.09 (1.08–1.10)	*<* *0.001*
Female sex	1.37 (1.20–1.57)	*<* *0.001*	1.19 (1.04–1.37)	* 0.01*	0.97 (0.89–1.07)	0.56	0.95 (0.87–1.04)	0.28
BMI	0.93 (0.82–1.06)	0.278	1.00 (0.99–1.01)	0.74	0.88 (0.81–0.96)	* 0.004*	0.99 (0.98–1.01)	0.19
Chronic lung disease	2.54 (2.21–2.91)	*<* *0.001*	1.96 (1.70–2.25)	*<* *0.001*	1.95 (1.77–2.15)	*<* *0.001*	1.73 (1.56–1.91)	*<* *0.001*
Extracardiac arteriopathy	3.21 (2.83–3.640)	*<* *0.001*	2.06 (1.80–2.35)	*<* *0.001*	2.26 (2.07–2.47)	*<* *0.001*	1.87 (1.71–2.05)	*<* *0.001*
Diabetes mellitus	2.24 (2.01–2.51)	*<* *0.001*	1.73 (1.54–1.95)	*<* *0.001*	1.55 (1.43–1.68)	*<* *0.001*	1.52 (1.40–1.66)	*<* *0.001*
Serum creatinine	1.00 (1.00–1.00)	*<* *0.001*	3.19 (2.48–4.09)	*<* *0.001*	1.00 (1.00–1.00)	*<* *0.001*	3.06 (2.53–3.70)	*<* *0.001*
Unstable angina	1.43 (1.21–1.69)	*<* *0.001*	1.05 (0.86–1.29)	0.62	1.60 (1.43–1.79)	*<* *0.001*	1.14 (1.00–1.31)	0.06
Recent myocardial infarction	1.26 (1.12–1.41)	*<* *0.001*	1.14 (1.02–1.29)	* 0.03*	1.39 (1.29–1.51)	*<* *0.001*	1.22 (1.12–1.32)	*<* *0.001*
Emergency	1.69 (1.41–2.04)	*<* *0.001*	1.47 (1.17–1.84)	* 0.001*	1.85 (1.62–2.10)	*<* *0.001*	1.51 (1.29–1.76)	*<* *0.001*
LVEF < 50%	2.65 (2.38–2.96)	*<* *0.001*	2.11 (1.88–2.36)	*<* *0.001*	1.98 (1.83–2.15)	*<* *0.001*	1.67 (1.54–1.81)	*<* *0.001*
Prior cardiac surgery	2.12 (1.61–2.78)	*<* *0.001*	2.05 (1.20–3.50)	0.22	1.81 (1.49–2.20)	*<* *0.001*	1.84 (1.25–2.71)	* 0.002*
Off-pump CABG	0.87 (0.76–0.99)	* 0.041*	0.83 (0.73–0.96)	* 0.01*	0.79 (0.71–0.87)	*<* *0.001*	1.03 (0.87–1.21)	*<* *0.001*
≥ 2 arterial grafts	0.47 (0.41–0.54)	*<* *0.001*	0.76 (0.66–0.89)	*<* *0.001*	0.96 (0.82–1.12)	0.07	1.02 (0.87–1.21)	0.75

*BMI* body mass index, *CABG* coronary artery bypass grafting, *CI* confidence interval, *ECC* extracorporeal circulation, *HR* hazard ratio, *LVEF* left ventricular ejection fraction

### Reintervention

Reintervention rate in the total population of the study is 3.5% (*n* = 1792). A higher non-adjusted reintervention rate was found in women than in men (4.5% vs 3.2%, *p* < 0.001), which is mainly driven by a higher number of unplanned postoperative PCIs.

## Discussion

The aim of this study was to assess differences in early and mid-term outcomes between male and female patients undergoing CABG in the Netherlands, thereby determining the factors which contribute to these differences in outcomes. In this large population, we found that women had a higher 30-day and mid-term survival rate after CABG compared to men. Additionally, we found that, in the age subgroup < 70 years, female patients had a worse mid-term mortality. Factors associated with mid-term mortality were: age, chronic lung disease, PVD, DM, serum creatinine > 200 µm/l, recent MI, decreased LVEF and emergency surgery. For patients aged > 70 years, factors associated with worse mid-term survival were the same as the commonly known risk factors for a worse outcome after CABG [9, 10].

Similar results to ours have been described in a study of the national database of the United Kingdom [11]. Our current analysis confirms the considerations of previous studies, which suggested that differences in mortality between sexes may be due to a different preoperative risk profile, presentation of CAD, and in surgical techniques used [12]. The current study shows that the poorer mid-term survival of women is only observed in patients aged < 70 years.

Differences between women and men in the presentation of CAD have been extensively described in earlier studies. Chest pain is more common in women than in men. In women this pain is less frequently caused by atherosclerotic lesions in the major epicardial coronary arteries [13, 14]. Men more frequently suffer from three-vessel and left main disease. Due to the more diffuse character of CAD in women, with less obstructive CAD and more single-vessel disease, non-invasive tests are less accurate in diagnosing CAD [15]. This, then, could lead to a delay in referral, diagnosis and intervention for women and might cause women to present with unstable angina and in emergency settings. Further research is needed on this correlation.

In agreement with other studies, women in our population presented at an older age, and with more comorbidities. It has been suggested that this ‘more complex risk profile’ is explanatory for the worse outcomes after CABG observed in women [16, 17]. Contrary to this claim, several studies found that female sex is a predictor of worse outcome after CABG, even after risk adjustment and propensity-matched analysis [18, 19]. We found female sex to be an independent predictor of worse mid-term mortality, only in the subgroup of patients aged < 70 years. Younger women having a worse outcome after CABG compared to younger man has been described by Vaccarino et al. [19]. Hypothetically, younger women presenting with premature CAD could have sex-related risk factors or be lacking protective factors normally present in women [6]. The protective effect of oestrogen on the cardiovascular system has been thoroughly researched; it is thought to decrease atherosclerotic lesion formation and to improve endothelial function [13]. More diffuse CAD and coronary microvascular dysfunction in women could explain why CABG leads to a higher mortality rate in younger women, as older women have disease patterns more similar to the disease pattern of men and, therefore, they benefit equally from CABG [17].

An important finding of this study is that women were less likely than men to receive ≥ 2 arterial graftsn. Additionally, we found that the use of ≥ 2 arterial grafts was associated with better mid-term outcome after CABG. This finding is in line with a recent review article in which the use of multiple arterial grafts was associated with a better outcome as compared to the use of venous graft material in addition to a single arterial graft [20]. However, in our study population, the use of ≥ 2 arterial grafts was only associated with improved outcomes in patients aged < 70 years.

Women receiving fewer arterial grafts is a phenomenon that has been described in several other studies [21, 22]. An explanation for this observation could be that women are more likely to present in an emergency setting. Mickleborough et al. have demonstrated a decreased use of the left internal mammary artery and decreased multi-arterial use in emergency settings [21]. Arguments against using the radial artery in women include the possibility that their radial artery might be smaller [23]. Lawton et al. found that there was a significant difference in the size of the radial artery of women and men, even when indexed for a difference in body surface area [23]. A recent systemic review and meta-analysis did not find a difference in vein graft patency between men and women, which indicates that the use of arterial grafts in women is as important as it is in men, assuming that the incidence of saphenous vein graft failure is the same in men and in women because it has been shown that the patency is the same [24].

In agreement with earlier studies [25], the present study shows that women received fewer distal anastomoses than men. Complete revascularisation has been associated with a better long-term outcome after CABG [25]. It might be assumed that complete revascularisation is less frequently achieved in women because women have smaller and more diffusely diseased coronary arteries. This then could lead to a worse mid- and long-term outcome. Our findings confirm that the incidence of coronary reintervention is higher in women than in men, which could indicate that, before the reintervention, there is ongoing ischaemia from non-revascularised arteries. However, reintervention data of the NHR are not corrected for competing risks.

The use of the OPCAB technique is correlated with an improved mid-term survival, in both age subgroups. In both subgroups, OPCAB was used more frequently in women than in men. Several studies have described the benefit of OPCAB over ONCAB in women [26, 27]. Evidence for the benefits of OPCAB hase especially been shown in high-risk patients of both sexes [28]. Consequently, one of the reasons why women seem to benefit more from OPCAB might be that they have a higher preoperative risk when presenting for surgery. These findings call for further research into OPCAB surgery in women.

In addition to a higher late mortality rate, women also have a higher reintervention rate than men in our study population. This difference was particularly significant for unplanned PCI reinterventions. This finding is in line with the observations of Guru et al. [29]. However, after propensity matching and risk adjustment, the difference in the number of reinterventions between the sexes was no longer seen in their study. Other investigators have reported that women are more likely to present with recurrent angina pectoris postoperatively and that postoperative MI occurs more frequently in women [19, 30]. Unfortunately, recurrent postoperative angina is not included as an outcome in the registry data of the NHR. On the other hand, we found that postoperative MI occurred more often among women than among men.

### Limitations

The current study has some limitations mostly due to the retrospective nature of the study. Additionally, some variables, such as ECC and cross-clamp time, were missing in a relatively large proportion of our study population. The primary endpoint of the study is all-cause mortality. The registry does not include the cause of death, which could be relevant for the interpretation of mortality rates.

## Conclusions

This study is the first large multicentre study in the Netherlands analysing the differences in outcomes between male and female patients undergoing CABG. Early mortality was higher in women, but female sex itself was not associated with mid-term mortality. For patients aged < 70 years, female sex was associated with worse mid-term survival. Women were less likely to receive ≥ 2 arterial grafts and received a lower number of distal anastomoses.

## Supplementary Information

Table S1 Definitions according to the Netherlands Heart Registration

Table S2 Baseline characteristics per subgroup

Table S3 Cox regression analysis for mid-term mortality in the total cohort

## Data Availability

Data will be made available to interested investigators who submit a reasonable research request by e‑mail.
