# Supplementary material for: Sex difference in outcomes after coronary artery bypass grafting: follow-up data of the Netherlands Heart Registration
Source: Neth Heart J. 2024 Dec 16;33(1):26–33. doi: 10.1007/s12471-024-01920-5 (PMC11695514; doi:10.1007/s12471-024-01920-5)
Supplement: Supplementary file 1 — Table S1 Definitions according to the Netherlands Heart Registration [file 12471_2024_1920_MOESM1_ESM.docx]

**Table S1.** Definitions according to the Netherlands Heart Registration

**Perioperative myocardial infarction**

Increase and/or decrease in one or more cardiac biomarkers (preferably troponin) by at least

one value above the 99th percentile of the upper limit, in the presence of at least one of the following symptoms:

• Symptoms consistent with ischemia (chest pain; nausea/vomiting/perspiration; shortness of breath due to left ventricular failure; dizziness/lightheadedness/syncope)

• New significant ST-segment or T-wave abnormalities or bundle branch block

• Development pathological Q waves on the electrocardiogram

• Imaging new loss of viable myocardial tissue or new wall movement disorders

• Identification of intracoronary thrombus at angiography or autopsy.

**Pneumonia**

Lung infection/pneumonia with positive sputum cultures.

**Urinary tract infection**

Infection with positive urine culture.

**Reintubation due to respiratory insufficiency**

Respiratory failure requiring reintubation.

**Prolonged intubation (>24 hours)**

Ventilation for more than 24 hours.

**Readmission to ICU**

Readmission to the Intensive Care Unit (ICU) or Post Anesthesia Care Unit (PACU) after initial discharge from the IC/PACU. This does not include a stay in the Medium Care (MC).

**Stroke**

The combined endpoint of stroke without neurological deficit and stroke with neurological deficit.

**Stroke without neurological deficit**

A neurologist has determined that a central neurological deficit (CVA) during

the postoperative period has occurred, but with no residual injury at discharge.

This also includes a Transient Ischemic Attack (TIA)

**Stroke with neurological deficit**

A neurologist has determined that a postoperative stroke has occurred during the

hospitalization for the current intervention (excluding TIA).

CVA = permanent neurological dysfunction diagnosed by a neurologist as

due to focal ischemia of the brain, spinal cord, or retina, caused by

an acute infarction of the neurological tissue due to thrombosis, embolism,

systemic hypoperfusion or bleeding.

**Kidney failure**

Renal failure that occurs if one or more of the following STS criteria are met during the postoperative period

• Renal replacement therapy (dialysis, CVVH) which was not initiated preoperatively

• Highest postoperative creatinine value > 177 µmol/L and doubling of the preoperative value (preoperative value being the value of the creatinine used to calculated the EuroSCORE).

**Gastro-intestinal complications**

Bleeding: gastrointestinal bleeding requiring therapy such as transfusion,

scope or surgery.

Other: intestinal ischemia, acalculous cholecystitis.

**Vascular complications**

The occurrence of any vascular complications during hospitalization,

diagnosis according to the VARC-2 definitions, from the start of the current intervention

(including peroperative vascular complications and excluding stroke).

**New-onset arrhythmia**

All forms of de novo rhythm problems requiring treatment (such as

resuscitation in connection with asystole, new onset atrial fibrillation / flutter for which

specific intervention (defibrillation, medication) is necessary). This does not include: a spontaneously transient period of atrial fibrillation, without any

consequence for the patient.

**Mediastinitis**

Deep sternal wound infection (mediastinitis) within 30 days. Includes muscle, sternum,

mediastinum and is positive if one or more of the following criteria is present:

• Surgical drainage / sternum refixation in deep sternum wound infection

• Positive wound cultures.

• AB therapy due to the sternum wound.

This includes a deep sternal wound infection that occurred after the patient

was discharged from the hospital.

**Reexploration (within 30 days)**

Rethoracotomy within 30 days after initial surgery due to a complication of the current intervention. This also includes rethoracotomies performed after the patient has been discharged.

This concerns the first rethoracotomy after the initial closing of the thorax. This

applies to all causes, with the exception of opening the sternum in due to

mediastinitis or refixation of the sternum.
