# Supplementary material for: Sex difference in outcomes after coronary artery bypass grafting: follow-up data of the Netherlands Heart Registration
Source: Neth Heart J. 2024 Dec 16;33(1):26–33. doi: 10.1007/s12471-024-01920-5 (PMC11695514; doi:10.1007/s12471-024-01920-5)
Supplement: Supplementary file 2 — Table S2 Baseline characteristics per subgroup [file 12471_2024_1920_MOESM2_ESM.docx]

| **Table S2** Baseline characteristics per subgroup | | | | |  |
| --- | --- | --- | --- | --- | --- |
|  | Age < 70 | Age ≥ 70 | *P*-value |  |  |
|  | *N*=30,412 | *N*=21,341 |  |  |  |
| Age, years | 62.0 ± 6.9 | 75.4 ± 4.0 | **<0.001** |  |  |
| Female gender | 4,801 (15.8) | 5,247 (24.6) | **<0.001** |  |  |
| BMI | 27.7 [25.1 – 30.2] | 26.9 [24.6 – 29.1] | **<0.001** |  |  |
| BSA, m^2^ | 2.01 [1.90 -2.14] | 1.95 [1.82 - 2.05] | **<0.001** |  |  |
| Chronic lung disease | 2,592 (8.5) | 2,404 (11.3) | **<0.001** |  |  |
| Extracardiac arteriopathy | 2,934 (9.6) | 3,177 (14.9) | **0.001** |  |  |
| Diabetes | 7,419 (24.4) | 5,868 (27.5) | **<0.001** |  |  |
| Serum creatinine ,μm/l | 84 [73 - 95] | 89 [77 - 105] | **<0.001** |  |  |
| Unstable angina | 2,677 (8.8) | 2,049 (9.6) | **0.01** |  |  |
| Recent myocardial infarction | 9,975 (32.8) | 6,851 (32.1) | 0.10 |  |  |
| Emergency | 1,780 (5.9) | 1,345 (6.3) | **0.04** |  |  |
| LV function |  |  | **<0.001** |  |  |
| Good, | 21,016 (69.1) | 14,029 (65.7) |  |  |  |
| Moderate | 7,402 (24.3) | 5,903 (27.7) |  |  |  |
| Poor | 975 (3.2) | 773 (3.6) |  |  |  |
| Very poor | 225 (0.7) | 144 (0.7) |  |  |  |
|  |  |  |  |  |  |
| Prior cardiac surgery, n (%) | 596 (2.0) | 497 (2.3) | **0.01** |  |  |
| EuroSCORE I, median [IQR] | 1.77 [1.29 – 3.04] | 4.30 [2.47 -7.58] | **<0.001** |  |  |

Data are presented as mean ± SD, median [IQR] or n (%)

BMI: Body Mass Index, BSA: Body Surface Area, LV: left ventricular
