# Supplementary material for: Sex difference in outcomes after coronary artery bypass grafting: follow-up data of the Netherlands Heart Registration
Source: Neth Heart J. 2024 Dec 16;33(1):26–33. doi: 10.1007/s12471-024-01920-5 (PMC11695514; doi:10.1007/s12471-024-01920-5)
Supplement: Supplementary file 3 — Table S3 Cox regression analysis for mid-term mortality in the total cohort [file 12471_2024_1920_MOESM3_ESM.docx]

**Table S3** Cox regression analysis for mid-term mortality in the total cohort

|  |  | Univariate |  | Multivariate |  |
| --- | --- | --- | --- | --- | --- |
|  |  | HR (95%) | *P-*value | HR (95%) | *P-*value |
| Age |  | 1.08 (1.08 - 1.08) | **<0.001** | 1.07 (1.07 - 1.08) | **<0.001** |
| Female sex |  | 1.25 (1.16 - 1.34) | **<0.001** | 1.03 (0.95 - 1.11) | 0.45 |
| BMI |  | 0.99 (0.98 - 0.99) | **0.002** | 0.99 (0.99 - 1.00) | 0.52 |
| Chronic lung disease |  | 2.28 (2.10 - 2.47) | **<0.001** | 1.80 (1.66 - 1.95) | **<0.001** |
| Extracardiac arteriopathy |  | 2.81 (2.62 - 3.02) | **<0.001** | 1.94 (1.80 - 2.09) | **<0.001** |
| Diabetes mellitus |  | 1.84 (1.72 - 1.96) | **<0.001** | 1.59 (1.48 - 1.71) | **<0.001** |
| Serum creatinine > 200 μm/l |  | 5.15 (4.46 - 5.96) | **<0.001** | 3.16 (2.72 - 3.67) | **<0.001** |
| Unstable angina |  | 1.57 (1.43 - 1.73) | **<0.001** | 1.12 (1.00 - 1.25) | 0.05 |
| Recent myocardial infarction |  | 1.32 (1.24 - 1.41) | **<0.001** | 1.20 (1.11 - 1.28) | **<0.001** |
| Emergency |  | 1.79 (1.61 - 1.99) | **<0.001** | 1.49 (1.31 - 1.70) | **<0.001** |
| LVEF <50% |  | 2.29 (2.15 - 2.44) | **<0.001** | 1.82 (1.70 - 1.94) | **<0.001** |
| Prior cardiac surgery |  | 1.99 (1.69 - 2.33) | **<0.001** | 1.90 (1.39 - 2.60) | **<0.001** |
| Off-pump CABG |  | 0.82 (0.76 - 0.89) | **<0.001** | 0.83 (0.77 - 0.90) | **<0.001** |
| ≥ 2 arterial grafts |  | 0.42 (0.38 - 0.47) | **<0.001** | 0.88 (0.78 - 0.98) | **0.02** |

BMI: Body Mass Index; LVEF: Left Ventricular Ejection Fraction; Off-pump CABG: Off-pump coronary artery bypass grafting
